# Documented Bladder Volume-Guided Timing and First-Attempt Pediatric Uroflowmetry Process Adequacy: A Retrospective Workflow Cohort Study

**DOI:** 10.3390/jcm15176849

**Published:** 2026-09-04

**Authors:** Yusuf Atakan Baltrak, Hasan Deliağa, Burak Bal

**Affiliations:** 1 Department of Pediatric Urology, Adana City Training and Research Hospital, 01230 Adana, Turkey; 2 S.B.Ü. Bursa Yüksek İhtisas Eğitim ve Araştırma Hastanesi, 16310 Bursa, Turkey; drhasandeliaga@hotmail.com; 3 Department of Pediatric Surgery, Adana City Training and Research Hospital, 01230 Adana, Turkey; burak94bal@gmail.com

**Keywords:** bladder capacity, bladder scan, child, lower urinary tract dysfunction, uroflowmetry, voided volume

## Abstract

**Background/Objectives:** Pediatric uroflowmetry is volume dependent, and low-volume voids can yield recordings that require repetition or cannot be interpreted confidently. To evaluate whether documented bladder volume-guided timing was associated with first-attempt pediatric uroflowmetry process adequacy in children undergoing evaluation for suspected non-neurogenic lower urinary tract dysfunction. **Methods:** This single-center retrospective workflow cohort included 110 toilet-trained children aged 5–12 years who underwent uroflowmetry for suspected non-neurogenic lower urinary tract dysfunction. The exposure was classified from contemporaneous pre-test documentation as bladder volume-guided timing (*n* = 55) or standard urge-based timing (*n* = 55). Expected bladder capacity (EBC) was calculated as (age + 1) × 30 mL. The primary process outcome was first-attempt voided volume ≥ 50% EBC. Repetition after an inadequate first attempt was treated as a deterministic workflow consequence rather than an independent endpoint. Analyses were observational and effect estimates were interpreted as associations. **Results:** Adequate first-attempt voided volume was documented in 50/55 children (90.9%) with bladder volume-guided timing and 39/55 (70.9%) with standard urge-based timing (unadjusted risk ratio 1.28, 95% confidence interval [CI] 1.06–1.55; Newcombe risk difference 20.0 percentage points, 95% CI 5.3–34.0). After adjustment for age, baseline urgency score, and time since last void, the association remained (adjusted risk ratio 1.27, 95% CI 1.06–1.53; *p* = 0.011), and the model converged without numerical warnings. Using the age-specific lowest acceptable voided volume, adequacy occurred in 53/55 children (96.4%) versus 46/55 (83.6%) (adjusted risk ratio 1.15, 95% CI 1.01–1.31; *p* = 0.040). Repetition after an inadequate first attempt occurred in 9.1% versus 29.1% and was treated as a direct consequence of primary-threshold failure; it was not tested independently. Workflow-time measures were exploratory. **Conclusions:** Documented bladder volume-guided timing was associated with greater first-attempt process adequacy. Adjustment for age, baseline urgency score, and time since last void did not materially change the estimate. The association was attenuated but remained directionally consistent when the age-specific lowest acceptable voided volume was used. Because the timing rule deliberately targeted the same volume construct as the primary outcome, this finding does not establish improved diagnostic accuracy, clinical decision making, or patient outcomes. Retrospective exposure classification and routine documentation further preclude causal interpretation. Documented bladder volume-guided timing was associated with higher first-attempt achievement of a prespecified voided-volume threshold than standard urge-based timing. Because the pathway targeted the same volume construct used to define the primary outcome and was non-randomized, these findings are interpreted as hypothesis-generating workflow data rather than evidence of improved diagnostic accuracy or downstream clinical benefit.

## 1. Introduction

Uroflowmetry with post-void residual (PVR) measurement is a basic noninvasive investigation in toilet-trained children with lower urinary tract symptoms. It provides information on voided volume, flow rates, voiding time, and curve morphology. These measurements are volume dependent, and a low-volume study may produce misleading flow values or a curve that cannot be interpreted confidently [1,2,3].

Expected bladder capacity (EBC) is commonly estimated as (age + 1) × 30 mL in children aged 4–12 years [1,4]. A voided volume below 50% of expected capacity is frequently treated as a pragmatic adequacy threshold, although no single threshold captures habitual functional capacity in every child [3]. An inadequate first test may require additional fluid intake, waiting, and repetition, increasing clinic time and staff workload.

Uroflowmetry is usually initiated when the child reports a desire to void. Subjective urgency may not indicate adequate bladder filling, particularly in children with urgency, anxiety, or reduced functional capacity. Portable bladder scanners provide a rapid estimate of bladder volume without catheterization or ionizing radiation. Adult studies suggest that pre-void scanning can reduce non-evaluable uroflowmetry [5,6], whereas pediatric studies have mainly assessed measurement performance, repeat-test agreement, and the influence of age, volume, and patient position [7,8,9,10]. Recent work also emphasizes adequate voided volume and standardized curve documentation for reproducible pediatric interpretation [11,12,13,14].

The clinical value of using bladder volume information to guide pediatric uroflowmetry timing remains uncertain. Importantly, a volume-guided pathway may combine several active components, including scan-estimated bladder volume, structured waiting, controlled hydration, staff prompting, and decisions to defer or proceed with voiding. We therefore evaluated whether documented bladder volume-guided timing was associated with greater first-attempt process adequacy than standard urge-based timing. Because the timing pathway targeted the same volume construct used to define the primary outcome and pathway assignment was non-randomized, the study was framed as a retrospective process-improvement association rather than an assessment of diagnostic accuracy or downstream clinical benefit.

## 2. Materials and Methods

### 2.1. Study Design and Setting

This single-center retrospective workflow cohort study was conducted in a tertiary pediatric urology outpatient clinic and included all potentially eligible uroflowmetry visits identified in the electronic and unit records between January and June 2026. The report follows the Strengthening the Reporting of Observational Studies in Epidemiology (STROBE) and REporting of studies Conducted using Observational Routinely-collected health Data (RECORD) recommendations [15,16]. The institutional ethics committee approved the study (3 June 2026/0196).

### 2.2. Data Source and Participants

The electronic medical record, uroflowmetry database, bladder scanner records, and unit workflow logs were searched for potentially eligible visits. Toilet-trained children aged 5–12 years were eligible when uroflowmetry had been requested for suspected non-neurogenic lower urinary tract dysfunction, the timing strategy could be classified unambiguously from documentation entered before the first void, and first-attempt voided volume was available. Referral categories were suspected dysfunctional voiding, symptom-defined overactive bladder, recurrent urinary tract infection, and other non-neurogenic indications. The Dysfunctional Voiding Symptom Score (DVSS) recorded routinely from the child/caregiver before uroflowmetry was extracted as a baseline symptom-burden measure; higher scores indicate greater symptom burden.

Exclusion criteria were active symptomatic urinary tract infection, known neurogenic bladder, anatomical bladder outlet obstruction, previous lower urinary tract surgery or reconstruction, indwelling catheterization or clean intermittent catheterization, current diuretic therapy, a behavioral or cognitive condition preventing routine uroflowmetry, inability to obtain a technically reliable bladder scan, or absence of documented parental permission for research use.

The exposure was the timing pathway documented before the first uroflowmetry attempt. A visit was classified as documented bladder volume-guided timing when the contemporaneous pre-void note explicitly stated that scan-estimated bladder volume informed waiting, controlled fluid intake, or test timing. A visit was classified as standard urge-based timing when uroflowmetry was initiated on the basis of the child’s reported desire to void and the scan result, if recorded, was not documented as influencing the initial timing decision.

Exposure classification was independently reviewed by a second pediatric urologist using the prespecified contemporaneous-documentation rule. The second reviewer was blinded to the extracted uroflowmetry outcomes and audited all 110 included records. Initial agreement was 104/110 (94.5%), with an unweighted Cohen kappa of 0.89 (95% CI 0.81–0.98). Six disagreements were resolved by consensus after re-examination of the contemporaneous pre-void notes; no record remained ambiguous, and the final classification comprised 55 children with bladder volume-guided timing and 55 with standard urge-based timing. Records without sufficient contemporaneous documentation for unambiguous classification were ineligible, and none of the 124 screened records was excluded for this reason. There was no written allocation rule: routine pathway use could vary with clinician preference, calendar period, staffing, and immediate device availability, but these determinants were not recorded consistently enough for reconstruction. No matching, post hoc balancing, or outcome-based sampling was performed; the 55/55 distribution was verified as the complete eligible source cohort.

### 2.3. Expected Bladder Capacity and Timing Strategies

Expected bladder capacity was calculated as EBC = (age in years + 1) × 30 mL. The primary adequacy threshold was a first-attempt voided volume ≥ 50% EBC. A pre-void scan volume > 115% EBC was recorded as potential overfilling. Severe urgency was defined as a score ≥ 8 on a 0–10 scale.

Pre-void bladder volume was measured in the supine position using the BladderScan BVI 9400 (Verathon Inc., Bothell, WA, USA). Two consecutive measurements were obtained; when they differed by >15%, a third measurement was taken, and the mean of the closest two was retained.

With bladder volume-guided timing, uroflowmetry was preferably initiated when the estimated bladder volume was ≥50% EBC. When the volume was below this threshold and urgency was <8/10, the child waited and could receive 5–7 mL/kg water (maximum 300 mL), followed by repeat scanning after approximately 15 min. Children with severe urgency, discomfort, leakage risk, or a request to void proceeded without delay. Bladder volume was therefore used as a flexible guide rather than an absolute requirement.

With standard urge-based timing, uroflowmetry was initiated when the child reported a usual desire to void. When the first voided volume was <50% EBC, the unit used its routine waiting and hydration process before offering a repeat test. The pre-void scan value was retained for retrospective analysis but was not documented as influencing the initial timing decision.

### 2.4. Uroflowmetry, Workflow Measures, and Curve Review

Uroflowmetry was performed using a Uroscan uroflowmetry system (Aymed Medical Technology, Istanbul, Turkey) in a private, child-friendly room. Boys voided standing and girls seated according to their usual position. Voided volume, maximum flow rate (Qmax), average flow rate, flow time, voiding time, and curve tracing were recorded. PVR was measured within 5 min using the same bladder scanner.

Total completion time was defined as the interval from the first recorded scan to completion of an adequate or final completed study and was derived from recorded workflow timestamps. Active staff time was the cumulative hands-on time documented in unit workflow logs for scanning, instruction, hydration, repeat preparation, uroflowmetry, and PVR measurement. The same timestamp sources and abstraction definitions were applied to both timing pathways throughout the study period. Because these variables depended on routine documentation and were partly determined by whether repetition occurred, they were treated as exploratory workflow outcomes. Parent satisfaction was not retained as an outcome because it was recorded with a single nonvalidated item.

Technical interpretability was not retained as an outcome because it was derived from the unblinded routine clinical report.

For the exploratory reliability analysis, the curves were deidentified and independently assigned to one of five morphology categories (bell, tower, plateau, staccato, or interrupted) by two pediatric urologists who were unaware of timing strategy, scan volume, referral indication, and each other’s rating. The independent ratings were used to calculate percentage agreement and unweighted Cohen kappa; no adjudicated rating was used in the agreement calculation.

### 2.5. Outcomes

The primary process outcome was first-attempt voided volume ≥ 50% EBC. Repetition because of inadequate volume was reported only as the deterministic workflow consequence of failing the primary threshold, not as an independent efficacy endpoint. First-attempt voided volume and the voided volume/EBC ratio were continuous descriptions of the same construct. Exploratory outcomes were total completion time, documented active staff time, severe urgency, pretest urinary leakage, inability to void, and pre-void volume > 115% EBC. Blinded inter-rater morphology agreement was exploratory.

### 2.6. Data Extraction and Statistical Analysis

Data were extracted with a prespecified form, and the analytic dataset was checked for duplicate records, out-of-range values, internal inconsistencies, and the reported 55/55 pathway totals. All eligible records meeting the documented exposure and outcome requirements were included; therefore, no a priori sample size calculation was performed. No missing data were present for the outcomes reported in the analytic dataset.

Baseline distributions were described using standardized mean differences (SMDs), with absolute SMD values around 0.10 interpreted as small. Binary outcomes were summarized using risk ratios (RRs) and risk differences (RDs) with 95% confidence intervals (CIs). Two-sided Fisher’s exact tests were used for unadjusted binary comparisons, and Newcombe score intervals were used for RDs. Continuous outcomes were summarized as mean ± standard deviation or median [interquartile range] according to distribution. Welch independent-samples *t* tests were used for approximately symmetric continuous variables and Mann–Whitney U tests for skewed or ordinal variables. Mean differences were calculated as bladder volume-guided timing minus standard urge-based timing. Secondary analyses were not adjusted for multiplicity and were interpreted as exploratory.

For the primary association, a parsimonious modified Poisson model with robust standard errors was prespecified, with timing strategy as the exposure and first-attempt voided volume ≥ 50% EBC as the outcome. Age, baseline urgency score, and time since last void were selected a priori as clinically plausible confounders rather than by data-driven significance testing. Referral indication was not included in the primary model to preserve parsimony. The effect measure was the adjusted RR with its 95% CI and *p* value. A prespecified sensitivity analysis used the age-specific lowest acceptable volume (age × 5 + 50 mL). Both models converged without numerical warnings; robust standard errors were finite, and no extreme coefficient estimates were observed. Analyses were performed using Python 3.13.5 (Python Software Foundation, Beaverton, OR, USA) with SciPy 1.17.0 and statsmodels 0.14.6. Two-sided *p* < 0.05 indicated statistical significance; emphasis was placed on effect estimates and CIs rather than dichotomous significance.

## 3. Results

### 3.1. Cohort Selection and Baseline Characteristics

**Figure 1:**
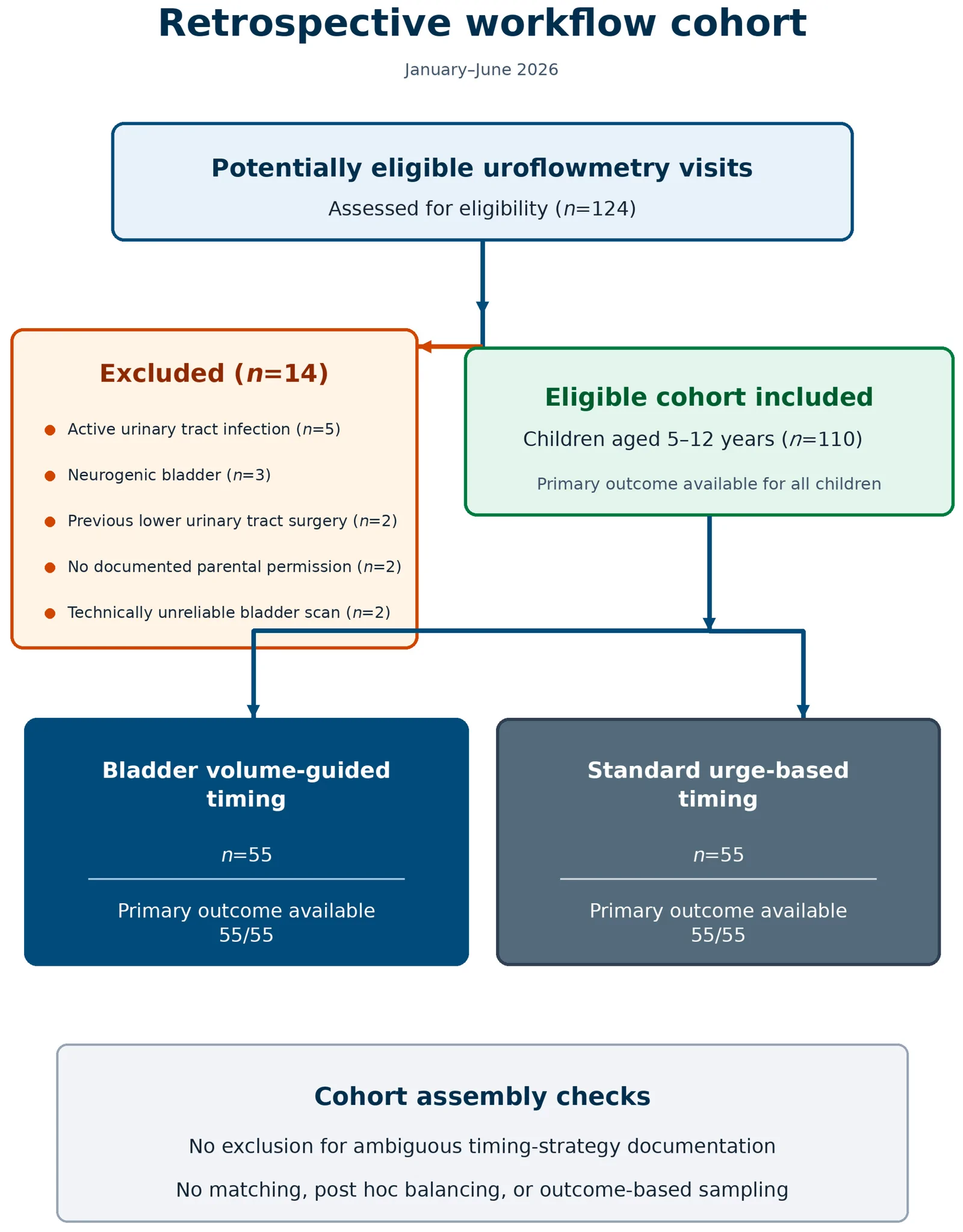
Flow of records through the retrospective workflow cohort. The pathway labels in the diagram correspond to bladder volume-guided timing and standard urge-based timing. No matching or post hoc balancing was performed.

All 124 potentially eligible uroflowmetry visits identified during the study period were assessed. Fourteen were excluded: active urinary tract infection (*n* = 5), neurogenic bladder (*n* = 3), previous lower urinary tract surgery (*n* = 2), absence of documented parental permission for research use (*n* = 2), and inability to obtain a technically reliable bladder scan (*n* = 2). No record was excluded for ambiguous timing-strategy documentation. The complete eligible cohort comprised 110 children: 55 with bladder volume-guided timing and 55 with standard urge-based timing. The equal group sizes arose from the verified source-cohort distribution; no matching, post hoc balancing, or outcome-based sampling was applied. Primary outcome data were available for all included children (Figure 1).

Measured baseline characteristics were similarly distributed, with absolute SMDs ranging from 0.00 to 0.10 (Table 1). This descriptive balance does not exclude unmeasured confounding or workflow-selection bias.

In the independent exposure-classification audit, the second reviewer agreed with the initial classification for 104/110 records (94.5%). The unweighted Cohen kappa was 0.89 (95% CI 0.81–0.98). All six disagreements were resolved by consensus after review of the contemporaneous pre-void notes, and the final pathway totals remained 55/55.

### 3.2. Primary Outcome

First-attempt voided volume ≥ 50% EBC was documented in 50/55 children (90.9%) with bladder volume-guided timing and 39/55 (70.9%) with standard urge-based timing (unadjusted RR 1.28, 95% CI 1.06–1.55; Newcombe RD 20.0 percentage points, 95% CI 5.3–34.0; Fisher’s exact *p* = 0.014). After adjustment for age, baseline urgency score, and time since last void, the adjusted RR was 1.27 (95% CI 1.06–1.53; *p* = 0.011). The model converged without numerical warnings, and robust standard errors and coefficient estimates were stable. Using the age-specific lowest acceptable voided volume, adequacy occurred in 53/55 children (96.4%) with bladder volume-guided timing and 46/55 (83.6%) with standard urge-based timing (adjusted RR 1.15, 95% CI 1.01–1.31; *p* = 0.040). Because this was an observational comparison, the reciprocal of the risk difference was not presented as a number needed to treat.

### 3.3. Directly Dependent and Exploratory Process Outcomes

Repetition after first-attempt inadequacy occurred in 5/55 children (9.1%) with bladder volume-guided timing and 16/55 children (29.1%) with standard urge-based timing. These were the same children who failed the prespecified first-attempt volume threshold. Therefore, repeat testing was reported only as a direct workflow consequence of primary-threshold failure; it was not tested independently or interpreted as an independent efficacy endpoint.

Mean first-attempt voided volume was 211 ± 71 mL with bladder volume-guided timing and 172 ± 76 mL with standard urge-based timing (mean difference 39 mL, 95% CI 11–67). The corresponding voided volume/EBC ratios were 75.2 ± 19.0% and 60.1 ± 22.7% (mean difference 15.1 percentage points, 95% CI 7.2–23.0). These continuous measures describe the same volume construct as the primary threshold and were not interpreted as independent confirmation. Total completion time (mean difference −8.8 min, 95% CI −14.9 to −2.7) and documented active staff time (−3.8 min, 95% CI −5.6 to −2.0) were exploratory and partly reflected the time added by repeat testing (Table 2).

### 3.4. Pre-Void Volume, Reliability, and Safety

At the initial scan, bladder volume was <50% EBC in 15/55 children (27.3%) with bladder volume-guided timing and 14/55 (25.5%) with standard urge-based timing (Fisher exact *p* = 1.00). Among the 15 bladder volume-guided children with an initially low volume, 12 reached a scan-estimated volume ≥ 50% EBC after additional waiting or controlled fluid intake, and 3 proceeded earlier because urgency was ≥8. In the complete cross-classification, 12/15 children (80.0%) with bladder volume-guided timing achieved adequate first-attempt volume and 3/15 (20.0%) did not, compared with 5/14 (35.7%) and 9/14 (64.3%), respectively, with standard urge-based timing. The exploratory subgroup RR was 2.24 (95% CI 1.06–4.73), and the Newcombe RD was 44.3 percentage points (95% CI 8.4–67.6; Fisher’s exact *p* = 0.025).

Overall agreement for blinded morphology classification was 84.5%; Cohen’s kappa was 0.78 (95% CI 0.69–0.87). Severe urgency, pretest leakage, inability to void, and potential overfilling were uncommon in both timing pathways (Table 3); the study was not powered to exclude clinically important differences in uncommon events. Additional exploratory technical, patient-centered, and device-agreement findings are provided in Table S1.

## 4. Discussion

In this retrospective workflow cohort, documented use of pre-void bladder volume to guide test timing was associated with a 20-percentage-point higher rate of adequate first-attempt uroflowmetry by the prespecified threshold. The adjusted estimate was similar to the unadjusted estimate (adjusted RR 1.27, 95% CI 1.06–1.53), while the alternative-threshold estimate was smaller but directionally consistent (adjusted RR 1.15, 95% CI 1.01–1.31). Repetition was the direct consequence of threshold failure, while continuous volume and workflow-time measures described related aspects of the same process. The primary finding therefore concerns process adequacy and does not constitute proof of improved diagnostic performance or clinical outcomes.

The proposed process mechanism is clinically plausible. Subjective urgency can occur before adequate bladder filling, particularly in children with urgency, anxiety, or reduced functional capacity. The almost identical proportions with an initially low scan-estimated volume are consistent with, but do not prove, a role for subsequent timing decisions. The active component may have been structured waiting and controlled hydration, with the scanner serving as a timing cue; this study cannot separate the device effect from that lower-cost workflow intervention. Clinician preference, calendar period, staffing, immediate device availability, and differential documentation may also have influenced pathway classification and outcome.

Previous adult studies used pre-void scan thresholds above the desired voided volume, recognizing that pre-void and voided volumes are not interchangeable [5,6]. Pediatric studies show that scanner error varies with age, bladder volume, and position; the BVI 9400 may underestimate supine pre-void volume, and individual error around a decision threshold can be clinically relevant [7,8,9]. Thus, a scan estimate close to 50% EBC does not guarantee a voided volume at the same threshold. The present study evaluates workflow rather than device accuracy, and no independent scanner-agreement analysis was retained.

Pediatric studies demonstrate observer variability and imperfect agreement between repeated uroflowmetry tests [10,11,12,13,14]. The separate blinded morphology review demonstrated substantial agreement, but it was exploratory and did not establish improved diagnostic performance.

The observed differences in completion and documented active staff time may be operationally relevant in high-volume clinics, but they were partly generated by the additional waiting and repetition that followed first-attempt inadequacy and were vulnerable to routine-log recording error. They are exploratory process descriptors, not independent efficacy findings, and they do not establish cost-effectiveness, diagnostic accuracy, treatment benefit, or improved downstream outcomes.

### Strengths and Limitations

Strengths include complete primary-outcome ascertainment, explicit documentation-based exposure rules, reporting of relative and absolute process estimates, and a blinded morphology review conducted separately from routine clinical interpretation.

The principal limitation is the non-randomized retrospective design. Routine pathway selection was not governed by a written allocation rule, and clinician preference, calendar period, staffing, immediate device availability, or patient factors may have produced selection bias and residual confounding. Although the 55/55 source-cohort distribution was verified and was not created by matching, measured baseline balance cannot address unmeasured confounding. Exposure classification relied on whether a contemporaneous note stated that scan volume influenced timing. The independent audit showed high agreement (94.5%; kappa 0.89), but the initial abstraction by the primary reviewer was not outcome-blinded, and differential documentation remains possible. Adjustment for age, baseline urgency score, and time since last void did not materially change the primary estimate, while the alternative-threshold association was attenuated. Neither analysis addresses unmeasured confounding or differential documentation. Workflow times were abstracted from routine logs. Because the primary threshold was embedded in bladder volume-guided timing, the study evaluates process adequacy rather than diagnostic utility; repeat testing and continuous volume measures are related consequences or alternative representations of the same event. Scanner error near the threshold and inability to separate scanning from structured waiting/hydration further limit mechanistic interpretation. The modest sample limited precision for uncommon events, multiple secondary analyses were exploratory, and the single-center, device- and operator-specific workflow limits generalizability.

## 5. Conclusions

In toilet-trained children undergoing uroflowmetry for suspected non-neurogenic lower urinary tract dysfunction, documented bladder volume-guided timing was associated with greater first-attempt process adequacy than standard urge-based timing after adjustment for measured confounders (adjusted RR 1.27, 95% CI 1.06–1.53). The association was attenuated but remained directionally consistent under the age-specific alternative threshold (adjusted RR 1.15, 95% CI 1.01–1.31). Because the pathway targeted the outcome construct and selection was non-randomized, the findings do not demonstrate improved diagnostic accuracy or patient benefit. A prospective multicenter comparison of bladder volume-guided timing with a standardized waiting-and-hydration pathway is warranted before routine implementation.

## Figures and Tables

**Table 1:** Baseline characteristics.

Characteristic	Bladder Volume-Guided Timing (*n* = 55)	Standard Urge-Based Timing (*n* = 55)	Absolute SMD
Age, years	8.4 ± 2.1	8.6 ± 2.0	0.10
Male sex, *n* (%)	31 (56.4)	30 (54.5)	0.04
Body mass index, kg/m^2^	17.5 ± 3.0	17.8 ± 3.2	0.10
Expected bladder capacity, mL	281 ± 63	287 ± 61	0.10
DVSS	10.3 ± 3.8	10.6 ± 3.9	0.08
Suspected dysfunctional voiding, *n* (%)	24 (43.6)	23 (41.8)	0.04
Symptom-defined overactive bladder, *n* (%)	17 (30.9)	18 (32.7)	0.04
Recurrent urinary tract infection, *n* (%)	8 (14.5)	8 (14.5)	0.00
Other non-neurogenic indications, *n* (%)	6 (10.9)	6 (10.9)	0.00
Baseline urgency score, 0–10	5.8 ± 2.0	5.7 ± 2.1	0.05
Time since last void, min	92 ± 38	89 ± 36	0.08

Data are mean ± standard deviation or *n* (%). DVSS, Dysfunctional Voiding Symptom Score; SMD, standardized mean difference. Baseline significance tests were not used. Similar measured distributions do not exclude unmeasured confounding or workflow-selection bias.

**Table 2:** Primary and exploratory workflow outcomes.

Outcome	Bladder Volume-Guided Timing (*n* = 55)	Standard Urge-Based Timing (*n* = 55)	Effect Estimate (95% CI)	*p* Value
First-attempt voided volume ≥ 50% EBC, *n* (%)	50 (90.9)	39 (70.9)	Unadjusted RR 1.28 (1.06–1.55); Newcombe RD 20.0 pp (5.3–34.0); adjusted RR 1.27 (1.06–1.53)	0.014 (unadjusted); 0.011 (adjusted)
Repeat after first-attempt inadequacy, *n* (%)	5 (9.1)	16 (29.1)	Deterministic consequence; no independent effect estimate	Not tested
First-attempt voided volume (same construct), mL	211 ± 71	172 ± 76	MD 39 (11–67)	Descriptive
Voided volume/EBC (same construct), %	75.2 ± 19.0	60.1 ± 22.7	MD 15.1 pp (7.2–23.0)	Descriptive
Total completion time, min	29.8 ± 13.5	38.6 ± 18.2	Exploratory MD −8.8 (−14.9 to −2.7)	Exploratory; 0.005
Active staff time, min	9.4 ± 3.1	13.2 ± 5.8	Exploratory MD −3.8 (−5.6 to −2.0)	Exploratory; <0.001

EBC, expected bladder capacity; RR, risk ratio; RD, risk difference; CI, confidence interval; MD, mean difference; pp, percentage points. Repetition after first-attempt inadequacy was reported descriptively because it was determined by failure to achieve the prespecified first-attempt volume threshold. It was not tested independently or analyzed as an independent efficacy endpoint. First-attempt voided volume and voided volume/EBC ratio describe the same volume construct as the primary process outcome.

**Table 3:** Adverse workflow events.

Outcome	Bladder Volume-Guided Timing (*n* = 55)	Standard Urge-Based Timing (*n* = 55)	Risk Difference, pp (95% CI)	*p* Value
Urgency score ≥ 8 before voiding, *n* (%)	7 (12.7)	6 (10.9)	1.8 (−10.8 to 14.5)	1.00
Pretest urinary leakage, *n* (%)	1 (1.8)	1 (1.8)	0.0 (−7.9 to 7.9)	1.00
Inability to void, *n* (%)	1 (1.8)	2 (3.6)	−1.8 (−10.6 to 6.4)	1.00
Pre-void volume > 115% EBC, *n* (%)	3 (5.5)	4 (7.3)	−1.8 (−12.4 to 8.6)	1.00

EBC, expected bladder capacity; CI, confidence interval; pp, percentage points. Confidence intervals for risk differences use the Newcombe score method. Fisher’s exact tests were used. The study was not powered for uncommon adverse events.

## Data Availability

Deidentified data are available from the corresponding author on reasonable request, subject to institutional and ethical restrictions.

## Supplementary Materials

The following supporting information can be downloaded at: https://www.mdpi.com/article/10.3390/jcm15176849/s1, Table S1. Exploratory technical, patient-centered, and device-agreement findings.
