# Supplementary material for: Documented Bladder Volume-Guided Timing and First-Attempt Pediatric Uroflowmetry Process Adequacy: A Retrospective Workflow Cohort Study
Source: J Clin Med. 2026 Sep 4;15(17):6849. doi: 10.3390/jcm15176849 (PMC13566094; doi:10.3390/jcm15176849)
Supplement: Supplementary file 1 [file jcm-15-06849-s001.zip › jcm-4500597-supplementary.pdf]

Supplementary Material

Association of Bladder Scan-Guided Test Timing With First-Attempt Uroflowmetry Adequacy in Children: A Retrospective Workflow Cohort Study

Supplementary Table S1. Exploratory technical, patient-centered, and device-agreement findings

Outcome	Scan-guided (n=55)	Standard (n=55)	Effect estimate / summary
Clinically documented interpretable first curve, n (%)	51 (92.7)	42 (76.4)	RR 1.21 (95% CI 1.03-1.43); P=0.033
Qmax, mL/s	18.7 ± 6.0	17.3 ± 6.3	MD 1.4 (95% CI -0.9 to 3.7); P=0.24
PVR, mL	18 [7-31]	20 [8-36]	Mann-Whitney P=0.49
Parent satisfaction, 0-10	8.8 ± 1.1	7.9 ± 1.6	MD 0.9 (95% CI 0.4-1.4); exploratory
Blinded morphology agreement	—	—	Agreement 84.5%; unweighted Cohen kappa 0.78 (95% CI 0.69-0.87)
Pre-void volume vs voided volume + PVR	—	—	ICC 0.89 (95% CI 0.84-0.92); mean bias +8.4 mL; 95% limits -35 to +52 mL

CI, confidence interval; ICC, intraclass correlation coefficient; MD, mean difference; PVR, post-void residual; Qmax, maximum flow rate; RR, risk ratio. Technical interpretability was abstracted from the original clinical report and was not assessed by blinded reviewers. Parent satisfaction was measured using a single nonvalidated item. The device-agreement comparison was not an independent accuracy validation because PVR was measured with the same scanner.
